# American Institutions for the Insane
*By Dr. Pliny Earll, in No. 32 of "The American Journal of Medical Science," edited by Dr. Hays :—1. Of the New York State Asylum, for 1851 and 1852.2. Of the New York City Asylum, for 1851 and 1852.3. Of the New Jersey State Asylum, for 1851 and 1852.4. Of the Pennsylvania State Hospital, for 1851 and 1852.5. Of the Frankford Asylum (Pa.), for 1851 and 1852.


**Published:** 1855-01-01

**Authors:** 


### AMERICAN INSTITUTIONS FOR THE INSANE.*

1. ALTHOUGH the report for 1854, by Dr. Benedict, of the New York State Lunatic Asylum, is less elaborate than that which preceded it, and a considerable portion of it occupied by an exposition of the necessity of new apparatus for heating the buildings, and other subjects of comparatively local interest, yet it furnishes us with some items of value in the physical department of the profession.

| | Men. | Women. | Total. |
|---|---|---|---|
| Patients at the commencement of the year . | 202 | 227 | 429 |
| Received in the course of the year . . | 185 | 181 | 366 |
| Whole number . . . . . . . . | 387 | 408 | 795 |
| Discharged, including deaths . . . | 167 | 193 | 360 |
| Remaining at the end of the year . . | 220 | 215 | 435 |
| Of the patients discharged, there were cured . | 58 | 54 | 112 |
| Died . . . . . . . . | 24 | 24 | 48 |

Applications for the admission of forty-seven patients, of whom sixteen resided in other States, were rejected.

The proportion of recoveries is smaller than usual, "for the reason," in the words of Dr. Benedict, "that we have been cautious in pronouncing a case recovered, though apparently well. We place all the cases of insanity from intemperance, from epilepsy, from general and gradual impairment of the faculties by age, and paroxysmal cases, though leaving the institution *well*, under the head of *improved* instead of *recovered*. The reason is obvious; there being no certainty that they will remain well for any length of time." This is "drawing the lines" a little closer, in regard to recoveries, than they have sometimes been drawn; as, for example, in an old report of one of our American asylums, in which one patient is recorded as "discharged—recovered," some six or eight times in the course of the year.

"The perfection and permanency of recoveries not unfrequently is cause of doubt and anxiety. Of the 1300 recoveries of the past nine years, 206 have been readmissions. Of the 51 readmissions of this year, 11 were persons who had been discharged well, in 1850. Two of these 11 were discharged recovered, in 1846 and 1847, one in 1847 and 1849, one in 1846, and two in 1849, making, in 11 persons, 20 recoveries, and 31 admissions."

The foregoing extract contains a detail which is too often neglected by the writers of these reports, but which is absolutely necessary to convey an accurate idea of the curability of insanity to the uninitiated reader.

Of the 112 cases of recovery, the duration of insanity before admission was one month and under, 36; two and three months, 44; four to seven months, 18; seven to twelve months, 9; over twelve months, 3; unknown, 2.

The time of residence at the Asylum, of the same cases, was—two months and under, 10; three months, 6; four to seven months, 54; seven to twelve months, 27; one to two years, 13; two years, 2.

"Dysentery, diarrhœa, and erysipelas," continues the report, "are the diseases with which we have to contend most frequently, *and when our ventilation shall be improved we hope to see these disappear*. We have had, during the

* By Dr. Pliny Earll, in No. 32 of "The American Journal of Medical Science," edited by Dr. Hays:—

1. Of the New York State Asylum, for 1851 and 1852.
2. Of the New York City Asylum, for 1851 and 1852.
3. Of the New Jersey State Asylum, for 1851 and 1852.
4. Of the Pennsylvania State Hospital, for 1851 and 1852.
5. Of the Frankford Asylum (Pa.), for 1851 and 1852.

year, 41 cases of dysentery, 25 males and 16 females. Duration of the disease varied from three to twenty days; average, nine days. One case in December, 1 in February, 3 in April, 1 in May, 1 in June, 2 in July, 23 in August, 10 in September. Fifty-five cases of diarrhœa; 23 males, 32 females; duration from two days to two months. These cases occurred during the severe months with those of dysentery, 39 of them in August. Twenty-four cases of erysipelas—9 males, 15 females; 3 of them were in December, 1 in January, 6 in March, 3 in April, 4 in May, 2 in June, 1 in August, 1 in September, and 3 in October. Average duration, eleven days. Six cases of typhoid, 3 of remittent, and 1 of intermittent fever. Acute affections of the lungs have been rare."

*Causes of death.*—Dysentery 6, diarrhœa 1, erysipelas 1, phthisis pulmonalis 11, chronic insanity 10, acute mania 1, general paralysis 2, epilepsy 5, pleurisy 1, malignant pustule 1, rheumatism 1, intemperance 1, suicide 5.

"The general prevalence of the suicidal propensity which was mentioned in my last report (and quoted in our former notices) as subsiding, returned with increased intensity, and continued throughout the winter and spring. In one case the act was committed soon after the admission of the patient, in whom there was no knowledge of the existence of the propensity. Another had been, during a residence of many months, remarkably cheerful and happy; an attack of erysipelas of the face confined him to bed, and rendered him very uncomfortable, and, at the height of the disease, he suspended himself from his window. All the suicides were by suspension from the window-bars, except one. To guard against such accidents, we have now adapted to a part of them sash-locks, which secure the windows from being opened and exposing the bars."

Statistics from the report for 1852:—

| | Men. | Women. | Total. |
|---|---|---|---|
| Patients at the commencement of the year . | 220 | 215 | 435 |
| Received in the course of the year . . | 200 | 190 | 398 |
| Whole number . . . . . . . . | 420 | 405 | 825 |
| Discharged, including deaths . . . | 205 | 195 | 400 |
| Remaining at the close of the year . . | 215 | 210 | 425 |
| Of those discharged, there were cured . | 92 | 64 | 156 |
| Died . . . . . . . . . . | 22 | 17 | 39 |

Sixty applications for admission were rejected.

"Of the 156 patients recovered, 92 are recorded *well*, and 64 *in usual health*. It may be proper to enter all these as *recovered*, they all having regained that state of mind possessed by them before their insanity; and yet many of them cannot be said to have that stability of character accompanying a sound mind. Under this head, *usual health*, we place that large class of weak-minded persons who run mad after every novelty, and again recover their equilibrium by seclusion in an asylum; and also others who leave apparently well, but are likely to become again deranged under exposure to the cause of previous attack. This division of recovered cases seems better than reporting the latter improved, as in our last report, which, in this respect, was a departure from established usage.

"The mortality for the past year is much less than for several previous years, while the amount of sickness has been about the same as last year. The principal diseases which prevailed during the year were: dysentery, 41 cases; diarrhœa, 45 cases, most of them in July and August; erysipelas, 21 cases; and typhoid fever 10."

*Causes of death.*—Phthisis pulmonalis 9, chronic insanity 6, epilepsy 5, phlegmonous erysipelas 4, opium-eating 3, dysentery 2, chorea 2, disease of heart 2, intemperance 2, acute dementia, general paralysis, apoplexy, and old age, 1 each. The number of deaths from acute disease is remarkably small.

"We are highly favoured in being able to report no deaths from suicide. This year only, since the second of the Institution's history, has passed without such an accident. Nor do we report any deaths from exhaustive mania ('typhomania,' 'phrenitis,' 'Bell's disease,' of other reporters). The number treated was eleven, some of whom had been greatly depleted previous to admission. We cannot urge our medical brethren too strongly to abstain from the practice of taking blood from insane persons. Our plan of treating very active insanity is directly opposed to depletion. Not one ounce of blood has been drawn from the 825 patients under treatment during the last year (fifty-four of these were of less duration than one month). We resort to stimulation in many cases with great freedom, and have seen the best evidence of its propriety."

| | |
|---|---|
| Patients admitted from Jan. 16, 1843, to Dec. 1, 1852 | 3499 |
| Discharged recovered | 1456 |
| Died | 407 |

To relieve the Institution from its most troublesome patients, such, too, as ought not to be associated with other insane persons, Dr. Benedict recommends "the erection of a hospital for 250 patients of the male sex only; to be carefully constructed, and fitted for the ultimate occupancy of lunatic criminals only; but to be used, until needed exclusively for this purpose, by criminal and homicidal lunatics, and drunkards." The suggestion is one well worthy of the attention of the public authorities in all the large States.

A scheme of moral treatment, including religious services, employment within doors and without, plays, tableaux, theatrical exhibitions, fairs, excursions, &c., is actively pursued. "The Opal," a magazine edited by the patients, is continued, and, by its more than three hundred exchanges, furnishes a great fund of transient reading matter, while the profits accruing from it during the year are sufficient to add several hundred volumes to a permanent library.

The legislature has appropriated thirty thousand dollars for the improvement of the means of heating and ventilating the buildings of the Asylum.

| | Men. | Women. | Total. |
|---|---|---|---|
| 2. At the New York City Lunatic Asylum, Blackwell's Island, the number of patients on the 1st of January, 1851, was | 200 | 264 | 464 |
| Admitted in course of the year | 216 | 225 | 441 |
| Whole number | 416 | 489 | 905 |
| Discharged, including deaths | 183 | 205 | 388 |
| Remaining, December 31, 1851 | 233 | 284 | 517 |
| Of those discharged, there were cured | | | 208 |
| Died | 37 | 43 | 80 |

Of the cases discharged, ten were delirium tremens, all cured.

*Causes of Death.*—Consumption 25, general debility 15, paralysis 11, chronic diarrhœa 8, epilepsy 5, apoplexy 4, dysentery 3, old age 2, pneumonia, phrenitis, carcinoma, hydrothorax, continued fever, gastritis, and albuminaria, 1 each. Of the patients admitted, 98 were natives of the United States, and 343 of foreign countries.

A considerable portion of Dr. Ranney's report is devoted to a history of the improvements of the Institution during the preceding five years—improvements, the result of which is that, "the very worst class of patients are as comfortably situated, at present, as were the best class in 1847."

Dr. R. suggests to the philanthropic a field for the useful employment of their benevolence, in taking charge of the poor insane, who, recovered from their mental disorder, are discharged from the Asylum without pecuniary means, or a place of employment. We most cordially "second that motion," and recommend it to the consideration of the benevolent in all places where there is a large institution for the insane, among the patients of which there are many from the poorer classes. Associations for the purpose alluded to have been formed in Europe, at Eberbach, in the Duchy of Nassau; at Stephansfeld, near Strasbourg; and at Vienna. It is said that they have been eminently useful.

Dr. A. V. Williams, one of the Visiting Physicians to this Asylum, resigned his place at the close of 1850.

Report for 1852:—

| | Men. | Women. | Total. |
|---|---|---|---|
| Patients, January 1, 1852 . . . . . | 233 | 284 | 517 |
| Admitted in course of the year . . . | 241 | 254 | 495 |
| Whole number . . . . . . . . | 474 | 538 | 1012 |
| Discharged, including deaths . . . | 248 | 237 | 485 |
| Remaining, December 31, 1852 . . . | 226 | 301 | 527 |
| Of those discharged, there were cured . . | | | 248 |
| Died . . . . . . . . . . | 70 | 60 | 130 |

Of the persons admitted, 102 were natives of the United States; 1, of Canada; 2, of Nova Scotia; 3, of Jamaica; and 387 of various European countries.

Of the cured, 10 were cases of delirium tremens; 3, of febrile delirium; and, 1, of typhus fever. The last two classes are placed under the head of *improper subjects;* as, also, are two cases of epilepsy, discharged *improved,* and four persons *not insane.*

*Causes of death.*—General debility 38, consumption 26, paralysis 15, typhus fever 10, diarrhœa 6, old age 5, paralysie générale 5, epilepsy 4, typhomania 3, apoplexy 3, mania 2, delirium tremens 2, dysentery 2, phrenitis 2, convulsions, pericarditis, laryngitis, pneumonia, erysipelas, and dropsy, 1 each.

The increase in the number of deaths over that of 1851 is attributed "almost entirely to the admission of improper subjects. In September, there were seven deaths of patients admitted within the month—all from long-standing diseases—not one of which ought to have been sent to the Asylum. The only endemic form of disease was from the 20th of November to the 15th of December. During this period twelve cases of typhus fever occurred, from which there were three deaths, one of this number being a highly valued attendant of the hall in which the disease originated. The only assignable cause for its production was a change of water. The main pipe for the conduction of the Croton water to the island having been broken, the supply was obtained from a well under one of the wings of the Asylum. On the re-introduction of the Croton, the disease disappeared."

"Two years and seven months have elapsed since a suicidal death occurred in this institution."

After a long struggle in the attempt to free the Asylum from penal convicts as attendants upon the patients, they have at length been entirely banished from the wards of the main building. "The experiment has proved," according to the report, "beyond all cavil, that this change has not increased the expense." Thus the great argument for the employment of such nurses has been effectually demolished. Yet, at the time the report was written, they were still employed in the "Lodge"—where are the apartments of the most violent patients—and in the kitchen, laundry, &c., of the main building. Dr. Ranney urges their entire removal for many plausible, and, at least to many persons experienced in the care of asylums, very obvious reasons.

The following case, of some importance in a medico-legal point of view, is mentioned in this report:—

"A patient, who committed homicide in the city, died last November. He became jealous of his wife, and killed the man whom he fancied was her paramour. The case was a remarkable one from the fact that, although he was actually insane at the time the deed was committed, yet, by the advice of a friend, he feigned another form of insanity. He believed that he had frequently seen Jesus Christ arise from the flame of a candle; that God had given him (who?) full power over the man (which?); but when examined, he pretended not to comprehend anything said to him, and for several weeks would only say, 'I don't know, sir.'"

3. Of the thirty-five pages of the annual report from the New Jersey State Asylum, for 1851, only six are occupied by that of its superintendent, Dr. Buttolph, and half of these are devoted to improvements made, and additions required, to the buildings.

| | Men. | Women. | Total. |
|---|---|---|---|
| Patients in Asylum, January 1, 1851 . . | 86 | 76 | 162 |
| Admitted in course of the year . . . | 50 | 52 | 102 |
| Whole number . . . . . . . . | 136 | 128 | 264 |
| Discharged, including deaths . . . | 51 | 42 | 93 |
| Remaining, January 1, 1852 . . . | 85 | 86 | 171 |
| Of those discharged, there were cured . . | 22 | 15 | 37 |
| Died . . . . . . . | 4 | 4 | 8 |

The number of patients in the course of the year was greater, by forty-four, than that of the preceding year.

Experience has proved that it is cheaper to light the buildings by gas, made upon the premises, than by oil.

From the nearly thirty pages of the essay upon the nature, forms, causes, means of prevention, and general principles of treatment of insanity, we cannot well make many isolated extracts. Nor are there many which would offer much novelty to persons who have already read Spurzheim or Combe. The following remarks upon attempts to define insanity are very just, irrespective of phrenology:—

"From this (the dependence of mental integrity upon the integrity of special physical organs) it will appear how utterly futile are attempts by physicians, physiologists, and jurists, to frame a definition of insanity so comprehensive as to embrace all supposable examples of the disease, and yet so particular as to be of practical utility in determining its existence in doubtful cases. Insanity, or mental derangement, being the opposite or counter state to sanity, or mental soundness; a knowledge of each individual standard of the latter must be had to enable us to exercise enlightened judgment of the existence and degree of the former in a given case. It may be remarked generally, therefore, that a state of insanity, or mental derangement, is that in which there is a departure, through disease of the brain, from the natural standard of thought and feeling of an individual, without his being conscious of the same, and in the loss of his ability to act freely in these circumstances. The expression of the sentiment embraced in this statement is deemed important, so far as it suggests the necessity, in each case, of a careful comparison of the supposed insane with the natural character of the individual, rather than a reliance upon a definition or rule of judgment that may not apply to his state or standard of mind.

"In criminal suits, involving the question of insanity, this rule or mode of procedure is quite as important to secure the ends of public justice, as to protect the rights of the culprit; because, conduct that would appear as the height of insanity in a majority of minds, may be in strict keeping with the standard of character in the person committing the offence, and indicate either an excusable degree of stupidity, or a most reprehensible state of depravity."

In regard to the question of isolation, we make the following extract:—

"When the mental derangement depends upon bodily disease of a temporary character, the patient should not be removed from home until a fair trial has been made for its cure; or, should it be very severe and more continued, he should not make the journey to an asylum under circumstances likely to increase it. Persons of advanced age, who are insane from the irregular decline of the faculties, or who are partially paralytic, but who have no dislike to their friends, and are quiet and manageable, may be as well treated at home as at a public institution. Again, very delicate females, who are only partially insane, but who cherish a strong attachment to home and friends, are sometimes unfavourably affected by the separation from them, and by association only with strangers. There may be yet other cases of this class, but there are more of which seclusion is of doubtful expedience, and can only be correctly determined by a careful consideration of all the circumstances attending them.

| | Men. | Women. | Total. |
|---|---|---|---|
| Patients in the Asylum, January 1, 1852 | 85 | 86 | 171 |
| Admitted in course of the year | 60 | 61 | 121 |
| Whole number | 145 | 147 | 292 |
| Discharged, including deaths | 54 | 56 | 110 |
| Remaining, January 1, 1853 | 91 | 91 | 182 |
| Of those discharged, there were restored | 19 | 26 | 45 |
| Died | 11 | 15 | 26 |
| Whole number from opening of the Asylum, May 15, 1848 | 264 | 251 | 515 |
| Discharged recovered | 81 | 80 | 161 |
| Died | 25 | 28 | 53 |

Twenty-five more cases were treated in 1852 than in any previous year.

The unusual number of deaths, the past year, was owing, "in part, to the great accumulation of chronic and enfeebled cases, and also from the occurrence of a dysenteric affection following the extremely hot weather of summer, and which proved fatal in nine instances of patients of this class." The other deaths were from congestion of the brain 3, epilepsy 4, consumption 4, chronic abscess, palsy, exhaustion, of acute mania, 1 each, and 3 from general exhaustion in dilapidated constitutions.

Dr. Buttolph urges the necessity of increasing the accommodations for patients by completing the original design of the building, in the erection of two additional wings. At one time, during the past year, the number of patients (208) was "more than benevolence would dictate, or than prudence would justify."

Stuart F. Randolph, Esq., a native of New Jersey, but for many years resident of New York city, has made a donation of two thousand dollars—with a pledge of five hundred more, should it be necessary—for the construction of a building, upon the Asylum grounds, to be used as a museum and reading-room by the patients.

4. After long, repeated, and persevering endeavours to establish a State Hospital for the Insane of Pennsylvania, success has at length been achieved —mainly through the untiring energy of Miss Dix—and we have the pleasure of bringing to the attention of our readers the first two reports of the superintendent of the institution, Dr. John Curwen.

We are informed in the first that the "Lunatic Hospital of the State of Pennsylvania" is situated about one and a half miles north of Harrisburg, upon a farm of one hundred and thirty acres. The corner-stone "was laid by Governor Johnston, on the 7th of April, 1849, and the building was delivered, by the architect and contractor, to the commissioners on the 19th of June, 1851." It "consists of a centre building, and a wing extending in a linear direction on each side. Each wing is so arranged that the second projection recedes twenty feet behind the first, and the third the same distance behind the second, so that the second and third projections are open at both ends, which renders them light and cheerful, and insures, at all times, a free, natural ventilation. The centre building is of three stories above the basement, or ground floor, has a large Tuscan portico, with a flight of twenty steps to the main entrance, and is surrounded (surmounted?) by a large dome, from which a very extensive view of the surrounding country is obtained."

The main wing, on either side, is of three stories, including the basement; the first receding portion three stories, and the second receding portion, intended for violent and noisy patients, two stories.

The whole building is warmed by air, heated, in the air-chambers in the basement, by steam passing through sixteen thousand feet of cast-iron pipes, which are connected with two cylinder boilers, each forty feet long and forty inches in diameter. It is lighted by gas, brought from the works of the Harrisburg Gas Company. It is abundantly supplied with water, and has, in its attic, four tanks of an aggregate capacity of twenty-two thousand gallons.

Thus, built upon one of the most approved models, and furnished with all the means which experience has proved to be most convenient and useful for an establishment of the kind, the institution was opened on the 1st, and received its first patient on the 6th of October, 1851. The number admitted between that time and the close of the year was thirty-seven. One was "a boy, six and a half years of age, whose disorder of mind was caused by convulsions during dentition, and who is yet subject to a slight spasmodic affection; and another a girl of thirteen years, in whom the mental derangement arose from epileptic convulsions, but, since a severe attack of bilious fever, six months ago, the convulsions have not returned." One epileptic patient had died.

We now proceed to the report for 1852.

| | Men. | Women. | Total. |
|---|---|---|---|
| Number of patients, December 31, 1851 . . | 24 | 13 | 37 |
| Admitted in the course of 1852 . . . . | 65 | 53 | 118 |
| Whole number . . . . . . . . | 89 | 66 | 155 |
| Discharged, including deaths . . . . | 29 | 19 | 48 |
| Remaining December 31, 1852 . . . . | 60* | 47 | 107* |
| Of those discharged there were cured . . | | | 13 |
| Died . . . . . . . . . . | | | 7 |

*Causes of Death.*—Exhaustion from acute mania, 1; paralysis, 1; latent pneumonia, 1; "exhaustion consequent on chronic mania," 4.

Dr. Curwen remarks:—

"Although the institution has been in operation more than a year, we have not yet found occasion to break through the rule which was adopted at the opening—never to use mechanical restraint, if it could by possibility be avoided. That cases have been received in which, by many, restraints would have been used, is freely admitted; but separation and seclusion for a few hours has generally accomplished the desired object, with much less irritation to the feelings of the patient, and less difficulty to the attendant."

The doctor's rule, however, literally interpreted, is somewhat too rigid. It admits of no exception. Mechanical restraints can, "by possibility," in every case, and always, be avoided. Yet there are patients to whom their application, even against the will of the party most immediately interested, is in accordance with, and the neglect of such application a dereliction from, the dictates of

* The report says 59 and 107—but such are not the results from the preceding data. The case of death by epilepsy, before the close of 1851, is probably retained in the number of patients at the beginning of the year.

true humanity; to say nothing of those who entreat their care-takers to bind their hands, lest they destroy the life of themselves or of persons around them.

The following remarks, if not novel, are nevertheless true:—

"I feel that I am discharging a part of my duty towards the insane, in calling attention to an error which is very extensively prevalent, and which consists in the almost invariable resort to bloodletting in all cases of insanity. All hospital experience, not only in this country, but also in Europe, has proved that the loss of blood, in any form of insanity, is almost uniformly attended with unpleasant effects, prolonging the period of cure, and, in many cases, placing the patient hopelessly beyond the reach of any benefit to be derived from subsequent treatment. Insanity is essentially a nervous disorder, and must be treated as such; and the greatest care should be taken to distinguish between that excitement which is purely nervous, and the delirium caused by inflammatory action. When any doubt exists, the abstraction of a few ounces of blood by cups or leeches, carefully watching the effects, will enable the physician to judge of the propriety of the course he is pursuing."

Let no one condemn this opinion of Dr. Curwen, on the ground that it is hastily formed, or based upon the experience of but little more than one year in a new institution. Previously to his connexion with the asylum at Harrisburg, he had been for several years the assistant physician of the Pennsylvania Hospital for the Insane.

The following extracts will show that the institution, even in its infancy, is as well supplied with the means of moral treatment as many have been in their adolescence, or manhood, and once more awaken our admiration for that benevolence, the copious current of which is now so freely running in the direction of the afflicted many who are suffering under psychic disorders.

"Religious services have been regularly maintained, on the Sabbath, during the whole year. * * * Evening prayers have also been regularly kept up during the year, to which all who feel inclined are invited to come.

"The Philadelphia fund for the benefit of the patients, collected through the instrumentality of that ardent friend of the insane, and of every benevolent work, Miss D. L. Dix, has already furnished essential advantages to the patients, in the use of a carriage and pair of horses, quite a number of musical instruments, two polyoramas, a large dioptric magic-lantern, with a number of slides, a small magic-lantern to be used in the wards, kaleidoscopes, backgammon-boards, and a number of other games, a large number of books, engravings, two large walnut book-cases, each capable of containing 250 volumes, and two globes. * * * A large piano, with an æolian attachment, has been ordered for the chapel. * * * A certain portion of this fund has been placed at my disposal for the erection of a reading-room and museum for each sex on the grounds of the institution."

5. The number of patients in the Frankford Asylum, on the 1st of March 1851, was 43; admitted during the year, 44; whole number, 87; discharged, including deaths, 35; remaining, March 1, 1852, 52. Of those discharged, 14 were cured and 6 died.

One died of fever, one of phthisis pulmonalis, one of exhaustion, one of effusion on the brain, and two by suicide.

"It is worthy of remark," says Dr. Worthington, "that the death from consumption is the first that has occurred among our patients from that disease for ten years past, especially as it is considered to be one of the most common causes of death among the insane.

"*Exhaustion* is a term which has been used to designate the cause of death in a peculiar form of disease, which appears to consist principally of intense excitement of the nervous system, with a tendency to rapid prostration of the vital energies, generally terminating fatally in the course of a few days."

It is well, when one uses a generic term with a specific signification, to explain its meaning, as Dr. Worthington has done in this instance. It is very evident that his "*exhaustion*" is nothing more nor less than the "typhomania," "phrenitis," "Bell's disease," "exhaustive mania," already alluded to. The same word, "exhaustion," is not unfrequently used by other reporters in, as we understand it, a very different sense. With them, we have always supposed it implied those cases of death in which there had been a gradual, but slowly-progressive wasting of the vitality of the body, without any evident, specific, organic lesion; in short, what other reporters still have called "marasmus"—a disease, by the way, which appears to have greatly diminished, since, although, according to the reports, it was in former years fatal to large numbers, not one case of death from it is mentioned in either of the reports reviewed in this article!

Dr. Worthington informs us that an unusually large number of suicidal patients were treated in the course of the year, and that six of them were cured.

One of the patients who died had been at the asylum about twenty-five years, and was 73 years of age; while, of the 52 remaining at the close of the year, 12 were upwards of 60. These facts, no less than that in regard to the exemption from pulmonary phthisis, are demonstrative of the salubrity of the location of the asylum, and of the excellence of the hygienic treatment.

On the 1st of March, 1852, there were 52 patients; admitted in the course of the year, 28; whole number, 80; discharged, including deaths, 24; remaining, at the close of the year, 56. Of those discharged, 15 were cured and 5 died.

"Of the five deaths, two were from phthisis pulmonalis, one of organic disease of the brain, one of epilepsy, and one of carditis. The two cases of consumption were admitted with that disease, and died, one eleven days, and the other about a year after entering the institution."

Since the opening of the institution, in 1817, "eleven hundred and sixty-nine patients have been admitted; of whom five hundred and fifteen have been discharged restored, and one hundred and fifty-five have died."

Although the report before us is well adapted to the persons for whom it is most particularly intended—those who have friends at the asylum, and those who are seeking the benefits of such an establishment—yet the subjects treated aside from those already noticed, furnish nothing of importance that would be new to our readers.

---

